# Microangiopathic Haemolytic Anaemia in a Young Male Patient With Oesophageal Carcinoma

**DOI:** 10.7759/cureus.17479

**Published:** 2021-08-27

**Authors:** Sebastian Ndlovu, Branislav Czako

**Affiliations:** 1 Trauma & Orthopaedics, Darent Valley Hospital, Dartford, GBR; 2 Haematology, Watford General Hospital, Watford, GBR

**Keywords:** oesophageal cancer, haemolytic uremic syndrome, thrombotic microangiopathy (tma), microangiopathic haemolytic anaemia, thrombotic thrombocytopenic purpura

## Abstract

Microangiopathic haemolytic anaemia (MAHA) in patients with various solid cancers and haematological malignancies has been reported, but to our knowledge, there has been no clearly reported case of MAHA in a young patient with oesophageal adenocarcinoma. MAHA is a subgroup of haemolytic anaemias characterised by destruction of red blood cells as they traverse small-calibre blood vessels. Its most defining features are anaemia and presence of fragmented red blood cells in the circulation. MAHA associated with cancer is now a well-recognized paraneoplastic syndrome, seen in various solid tumours and haematological malignancies, the most common being gastric, breast and lung carcinoma. The development of MAHA associated with any malignant process is usually an ominous condition, not only because of the fact that no convincing treatment has been discovered to date, but also because it invariably almost always occurs in disseminated cancers as a late presentation.

The prompt identification of the signs and symptoms suggestive of intravascular haemolysis, the deliberation of the cause of such symptoms and the concurrent ruling out of related conditions which may mimic MAHA symptoms such as haemolytic uremic syndrome and thrombotic thrombocytopenic purpura are crucial to ensure successful treatment. The patient is a 33-year-old male patient of Asian descent who had oesophageal adenocarcinoma that had metastasized to the peritoneal cavity and para-aortic lymph nodes. The patient was admitted with bilateral extensive deep vein thrombosis, and was later found to have pulmonary embolism as well. A few days after his admission, he suddenly developed shortness of breath, severe chest pain and was diagnosed with cancer-associated MAHA. His sudden, rapid clinical deterioration, and the inability to intervene successfully was a traumatizing experience for his doctors and relatives alike.

## Introduction

Microangiopathic haemolytic anaemia (MAHA) refers to mechanical haemolytic anaemia characterized by red blood cell fragmentation as they traverse small-calibre blood vessels, typically seen as schistocytes on peripheral blood smear [1]. MAHA is observed in numerous conditions such as haemolytic uremic syndrome (HUS), thrombotic thrombocytopenic purpura (TTP), systemic infection, disseminated intravascular coagulation (DIC), and immune disorders [2]. MAHA that occurs in patients with disseminated malignancies is now a recognized, rare paraneoplastic syndrome associated with various solid tumours and haematological malignancies, the most common being gastric, breast and lung carcinoma [2,3]. In cancer-related MAHA, MAHA is usually detected at initial diagnosis of cancer, but can also be associated with cancer recurrence. The diagnosis of MAHA in cancer patients invariably signifies end-stage disease with poor outcomes [3]. Clinical vigilance is necessary in all cases to avoid inappropriate management. 

## Case presentation

This is a case of a 33-year-old patient who presented with a four-week history of swelling of his left leg, which had acutely worsened over the preceding day and his right leg was getting involved as well. His left leg was also acutely painful such that he could no longer weight bear. These symptoms were associated with dysphagia, loss of weight (about 3 kg over four months) and drenching night sweats. He had no family history of malignancy, but his sister had a history of recurrent miscarriages. He had no other significant family history and no recent history of travel or long flights. He was a non-smoker, did not take alcohol, and was previously fit and well with no known allergies. He had been seen by his General Practitioner twice before presentation and started on non-steroidal anti-inflammatory drugs with a working diagnosis of reactive or inflammatory arthritis. On examination he had no enlarged lymph nodes; he had bilateral lower limb swelling up to thigh level, left more than the right and bilateral calf tenderness, but no signs of compartment syndrome.

**Figure 1:**
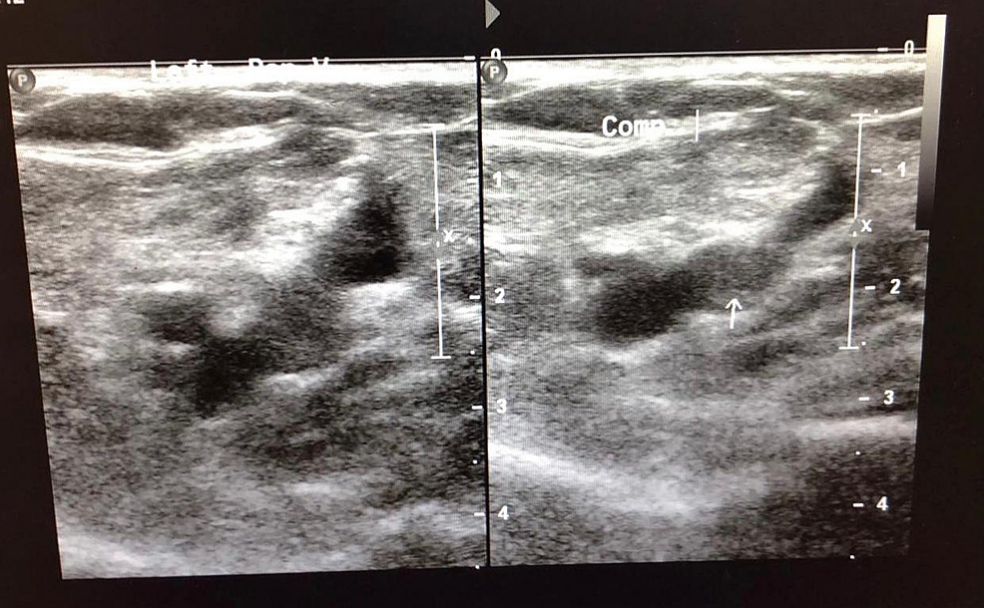
Doppler ultrasound scan image showing thrombus in left popliteal vein

**Figure 2:**
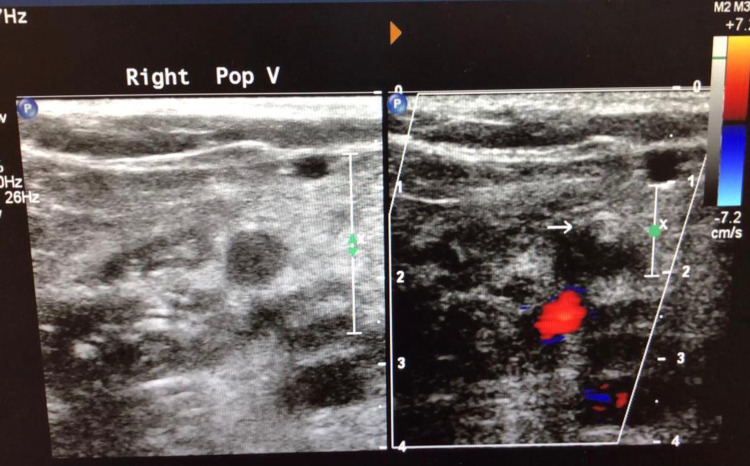
Colour Doppler ultrasound scan image showing thrombus in right popliteal vein

A full blood count on admission showed a mild normochromic normocytic anaemia with haemoglobin of 123 g/L, a haematocrit of 35% and a normal platelet count at 154 X 10^9^/L (previous results a month before this presentation were haemoglobin 136 g/L, haematocrit 41% and platelet count of 266 X 10^9^/L). The rest of the results revealed elevated D-dimers at 16,606 ng/mL, normal urea and electrolytes, mildly elevated prothrombin time (PT) at 14.8 s, normal activated partial thromboplastin time (APTT), low fibrinogen of 1.1 g/L and a normal serum bilirubin. An ultrasound Doppler performed showed bilateral deep vein thrombosis (Figure 1 and Figure 2) and he was put on a therapeutic dose of low-molecular-weight heparin (clexane). The differential diagnosis of thrombophilia was being entertained; however, paroxysmal nocturnal haemoglobinuria screen was negative, but lupus anticoagulant screen was positive and anti-cardiolipin antibodies were negative.

**Figure 3:**
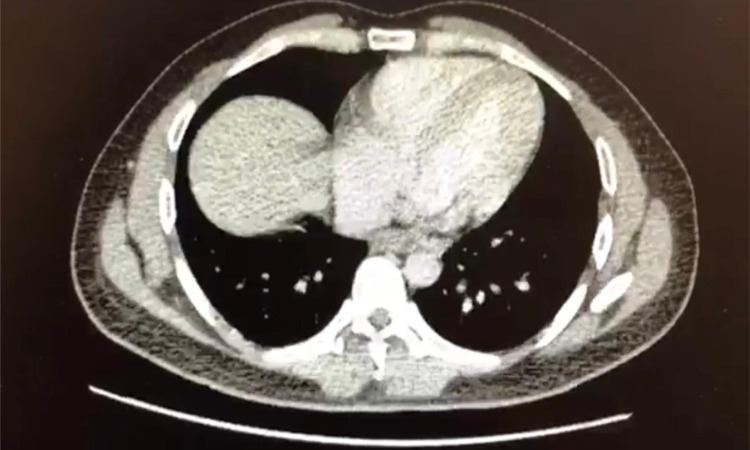
CT chest image showing bilateral segmental pulmonary arterial emboli

**Figure 4:**
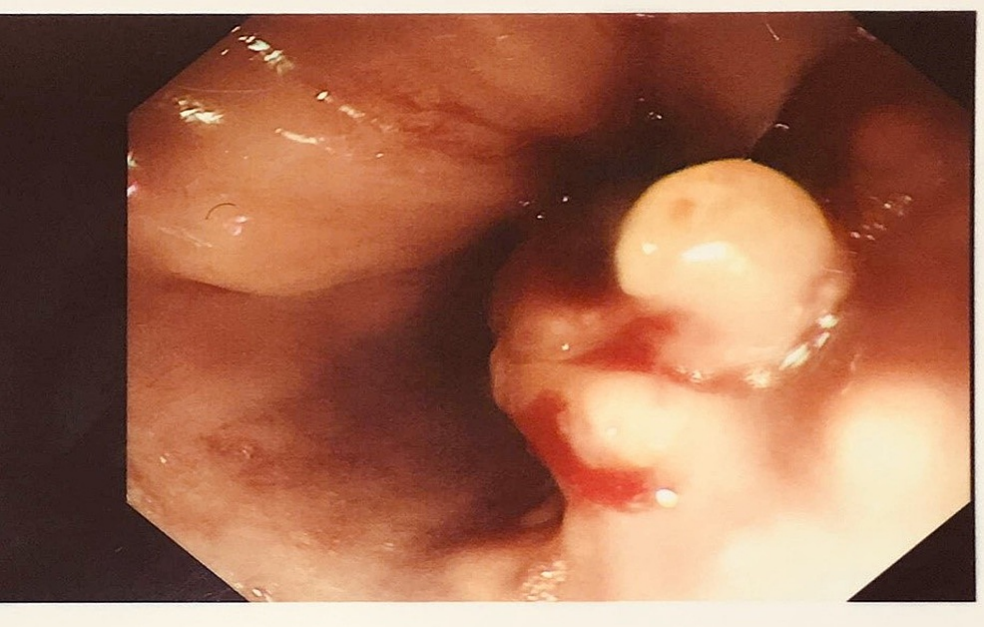
2 cm lesion noted in distal oesophagus during oesophagogastroduodenoscopy

A CT CAP (chest, abdomen and pelvis) scan done three days after admission to rule out malignancy as a cause of his symptoms showed multiple right segmental pulmonary arterial emboli, with a thrombus within the inferior vena cava (IVC) and enlarged subcarinal and para-aortic lymph nodes measuring up to 1.9 cm (Figure 3). Diagnostic laparoscopy was arranged as a diagnostic and staging tool, during which omental biopsies were taken and peritoneal washings performed. Findings included a much-thickened lesser omentum, reported to be highly suggestive of upper gastrointestinal (GI) malignancy. The biopsies would later reveal infiltration by poorly differentiated adenocarcinoma and cytology report from peritoneal washings showed cohesive clusters of atypical cells with pleomorphic nuclei consistent with a metastatic adenocarcinoma. A day later the patient had an oesophagogastroduodenoscopy, which showed a small polypoid oesophageal tumour, from 37 to 39 cm from teeth, but not extending into stomach (Figure 4). Several samples were taken, which also later revealed an oesophageal mucosa infiltrated by poorly differentiated adenocarcinoma. 

Two days after the diagnostic laparoscopy, the patient developed chest pain, pyrexia and shortness of breath and started desaturating on room air; however, his repeat CT pulmonary angiogram showed no new pulmonary embolism. He was stable on low-flow oxygen by nasal cannula, his blood pressure was normal, but he was borderline tachycardic. He had bi-basal crackles and was started on intravenous antibiotics for probable chest sepsis. His full blood count showed a massive decrease in his haemoglobin levels to 80 g/L, with a haematocrit of 22% and a reticulocyte of 245 X 10^9^/L (8.3%) but his platelet count was still borderline normal. Despite receiving 2 units of blood, his haemoglobin levels after transfusion were even lower at 69 g/L with a haematocrit of 20% and a platelet count of 122 X 10^9^/L. The direct antigen test was negative, and no atypical red cell antibodies were detected. Lactate dehydrogenase (LDH) was raised at 1307 U/L, and serum haptoglobin decreased to <0.1 g/L (NR: 0.5-2.0 g/L). His clotting profile was largely unchanged from the one done at admission. His serum bilirubin levels had increased to 83 umol/L with the unconjugated portion being 52 umol/L. His gamma-glutamyl transferase was also moderately raised to 135 U/L but the rest of his liver function tests were largely normal. An ultrasound scan of his biliary tract was done to rule out cholestasis, and it showed a normal biliary tree and common bile duct calibre. His renal function remained normal throughout his admission. ADAMTS 13 activity was borderline abnormal expressed as 63 IU/dL (NR 64-132 IU/dL). His blood film showed polychromasia, nucleated red blood cells (NRBCs), stomatocytes, schistocytes, anisocytosis, and genuine thrombocytopenia consistent with known cancer-associated MAHA.

His clinical condition deteriorated on the 10th day of his admission; he was feeling dizzy, short of breath and had increased oxygen demand. He was also having haematuria and was severely thrombocytopenic. The plan was to treat aggressively with fluid resuscitation, stop clexane and arrange for an IVC filter. Plasma exchange (PEX) has been discussed with a tertiary centre; however, it was declined because it is ineffective in cancer-associated MAHA patients. Despite receiving a total of six units of blood transfusion over three days and one unit of platelets, his haemoglobin and platelet levels never improved. His condition continued deteriorating and a decision was made to treat palliatively in consultation with a haematologist and intensive care physicians. Unfortunately, attempts to treat conservatively failed and he died 10 days after he was admitted.

## Discussion

Paraneoplastic syndrome is a collective term for disorders arising from metabolic effects of cancer on tissues remote from the tumour; such disorders may, for example, appear as primary endocrine, hematologic or neuromuscular disorders [4]. MAHA is defined by evidence of haemolysis (increased serum indirect bilirubin and decreased haptoglobin), fragmented RBCs and a negative direct antiglobulin (Coomb's) test [5]. Cancer-associated MAHA generally occurs as a rare and abruptly presenting paraneoplastic phenomenon [6]. The most common clinical features associated with cancer-associated MAHA were characterised by the Oklahoma TTP - HUS Registry as older age, male gender, gradual onset of symptoms that include weakness, weight loss, pain and respiratory symptoms [7]. Among the solid cancers, gastric, breast, prostate, lung and cancer of unknown primary are the most commonly associated ones with MAHA, with the majority (over 90%) being metastatic. MAHA is also associated with haematological malignancies including Hodgkin disease, angiotropic lymphoma, diffuse large cell lymphoma and myeloma [3].

The diagnosis of MAHA is a fairly easy one to make for an experienced haematologist using common laboratory tests, and these typically show decreased haemoglobin levels, reticulocytosis, thrombocytopenia, an elevated LDH, increased total bilirubin, decreased serum haptogloin and a negative direct Coomb’s test. The levels of fibrinogen and fibrin degradation products are usually elevated. Peripheral blood smear shows numerous fragmented erythrocytes (schistocytes), polychromasia, NRBCs and leukoerythroblastic reaction. MAHA is observed in various conditions such as TTP, HUS, DIC, systemic infection and immune disorders [8]. Some pathologists prefer the term thrombotic microangiopathies (TMAs) to describe these clinical syndromes. TMAs represent a heterogeneous group of diseases characterized by MAHA, peripheral thrombocytopenia and organ failure of variable severity. TMAs encompass TTP, typically characterized by fever and central nervous system manifestations, HUS in which renal failure is the prominent abnormality [2], and atypical haemolytic-uremic syndrome (aHUS), a genetic, life-threatening, chronic disease of complement-mediated TMA, which should be distinguished from the more common typical HUS [9].

The formulation of clinical differentials in patients with MAHA is imperative not only because of the risk of missing an underlying malignancy, but also because cancer-associated MAHA and TTP are oncological and haematological emergencies, respectively, in their own right. Patients with cancer-associated MAHA typically present with severe weight loss, dyspnoea, bone pain, as well as DIC and massive erythromyelemia, and, in contrast, these features are observed with a much lower incidence in patients with an idiopathic TMA [2]. Patients with Shiga toxin-mediated HUS caused by enteric infection with a Shiga toxin-secreting organism, such as *Escherichia coli* O157:H7, may present with gastrointestinal symptoms and obtaining stool cultures will be crucial in reaching a diagnosis. Differentiating TMAs from DIC is fairly straightforward, as such patients also have prolonged PT and APTT, decreased fibrinogen levels and elevated levels of fibrin degradation products in addition to thrombocytopenia.

The measurement of ADAMTS13 activity is appropriate for all patients presenting with MAHA and thrombocytopenia [5]. ADAMTS13 is primarily synthesized in the liver, and its main function is to cleave von Willebrand factor (VWF) anchored on the endothelial surface, in circulation, and at the sites of vascular injury. Deficiency of plasma ADAMTS13 activity (<10%) resulting from mutations of the ADAMTS13 gene or autoantibodies against ADAMTS13 causes hereditary or acquired (idiopathic) TTP [10]. The ADAMTS13 activity assay has a sensitivity and specificity of 100% and 99%, respectively, with a positive predictive value of 91% and negative predictive value of 100% using the 20% activity level cut-off and appropriate exclusions for interfering conditions [11]. It is an important diagnosis to make because the untreated mortality is 90%, which can be reduced with the prompt delivery of PEX. Diarrhoea positive (D+) HUS, on the other hand, is treated with supportive care, which in some cases includes renal dialysis [12]. Eculizumab, a humanised monoclonal antibody, inhibits complement-mediated TMA and was reported to cause improvement in renal function in patients with aHUS [9].

The pathogenesis of cancer-associated MAHA is poorly understood, as it appears to differ from primary TMA syndromes such as hereditary and immune-mediated TTP and HUS. In solid cancers, it has been hypothesized that the likely cause may be red cell fragmentation and platelet destruction in small vessels of cancerous tissue in the bone marrow, the lung and/or other organs. This, however, does not explain the absence of MAHA in most patients with the same metastatic pattern, and the fact that MAHA still occurs in patients with little or insignificant bone marrow infiltration or fibrosis. It has also been theorised that cytokine production by tumour cells may play a role, which may lead to direct or indirect endothelial cell damage [3]. Others have suggested that despite conflicting data, the presence of high levels of vWF multimers and a functional deficiency of ADAMTS13 cleaving protease may contribute to the pathogenesis of MAHA [6]. In patients with cancer and on treatment, chemotherapy may cause TMA by two mechanisms, namely an acute immune-mediated reaction or dose-dependent toxicity. Acute and presumed immune-mediated TMA has been reported with oxaliplatin, and other drugs associated with MAHA include 5-fluorouracil, bleomycin, cisplatin, cytosine arabinoside, daunomycin, deoxycoformycin, estramustine, methyl-CCNU, gemcitabine and the most commonly implicated mitomycin C. Mechanisms of pathophysiology are thought to include direct endothelial damage, platelet activation and uncontrolled alternative complement pathway activation [13].

Cancer can confer a prothrombotic or hypercoagulable state through an altered balance between the coagulation and fibrinolytic systems. TF (thromboplastin) is a 47-kDa transmembrane glycoprotein that initiates the extrinsic pathway of the coagulation cascade. It is a cellular procoagulant expressed in cells, including endothelial cells and monocytes-macrophages produced in response to proinflammatory stimuli such as the cytokines interleukin 1β (IL-1β), tumour necrosis factor α (TNF-α) and bacterial endotoxins. However, in contrast to normal cells, malignant cells express TF constitutively and thus have constant procoagulant activity (PCA).

Cancer procoagulant is a cysteine protease of 68 kDa, which can activate the common pathway of the coagulation cascade independently of FVII. Tumour cells can also express everything required for regulation of the fibrinolytic pathway on their cell surface. They possess both the urokinase-type and the tissue-type plasminogen activator and can also produce plasminogen activator inhibitor-1 and plasminogen activator inhibitor-2. Tumour cells also produce and secrete a number of different proinflammatory cytokines, some of which can adversely affect the normal anticoagulant system in the vascular endothelium. For example, TNF-α and IL-1β can induce the expression of TF by vascular endothelial cells and downregulate the expression of thrombomodulin, the surface receptor for thrombin leading to reduced activation of the protein C system, which is one of the main anticoagulant defence systems [14]. Whether or not this persistent PCA contributes to the development of MAHA is largely unknown, and possibly conjectural. It also remains to be defined whether a specific trigger may be responsible especially in this case, whereby MAHA occurred suddenly rather than the usual insidious variety. The existence of a proven hypercoagulability state (extensive DVT) and a possible trigger (surgical procedure, anaesthetic drugs), or some other factor not yet identified could have rapidly accelerated clinical deterioration. Regardless of the cause, cancer-associated MAHA has been shown to have a poor response to PEX; however, chemotherapy has been shown to be helpful in the short term [15].

## Conclusions

The clinical presentation of TMAs in cancer is a rare, complex clinicopathological entity that is largely not completely understood. Despite the confusion with regard to the pathophysiological process behind cancer-associated MAHA, prompt diagnosis and exclusion of the readily treatable differentials are necessary to mitigate the ominous outcomes. The poor response to PEX and conservative or supportive management means that chemotherapy should be the mainstay of treatment for cancer-associated MAHA. Some studies have reported that it improves haematological parameters, although overall prognosis is still poor with a median survival of three months with chemotherapy.
